# Efficient IoT-Assisted Waste Collection for Urban Smart Cities

**DOI:** 10.3390/s24103167

**Published:** 2024-05-16

**Authors:** Sangrez Khan, Bakhtiar Ali, Abeer A. K. Alharbi, Salihah Alotaibi, Mohammed Alkhathami

**Affiliations:** 1 Department of Electrical Engineering, École de Technologie Supérieure, Montréal, QC H3C 1K3, Canada; sangrez.khan.1@ens.etsmtl.ca; 2 Department of Electrical and Computer Engineering, COMSATS University Islamabad, Islamabad 45550, Pakistan; bakhtiar_ali@comsats.edu.pk; 3 Information Systems Department, College of Computer and Information Sciences, Imam Mohammad Ibn Saud Islamic University (IMSIU), Riyadh 11432, Saudi Arabia; sfosimi@imamu.edu.sa (S.A.); maalkhathami@imamu.edu.sa (M.A.)

**Keywords:** waste collection, waste management, smart city, IoT

## Abstract

Waste management is one of the many major challenges faced by all urban cities around the world. With the increase in population, the current mechanisms for waste collection and disposal are under strain. The waste management problem is a global challenge that requires a collaborative effort from different stakeholders. Moreover, there is a need to develop technology-based solutions besides engaging the communities and establishing novel policies. While there are several challenges in waste management, the collection of waste using the current infrastructure is among the top challenges. Waste management suffers from issues such as a limited number of collection trucks, different types of household and industrial waste, and a low number of dumping points. The focus of this paper is on utilizing the available waste collection transportation capacity to efficiently dispose of the waste in a time-efficient manner while maximizing toxic waste disposal. A novel knapsack-based technique is proposed that fills the collection trucks with waste bins from different geographic locations by taking into account the amount of waste and toxicity in the bins using IoT sensors. Using the Knapsack technique, the collection trucks are loaded with waste bins up to their carrying capacity while maximizing their toxicity. The proposed model was implemented in MATLAB, and detailed simulation results show that the proposed technique outperforms other waste collection approaches. In particular, the amount of high-priority toxic waste collection was improved up to 47% using the proposed technique. Furthermore, the number of waste collection visits is reduced in the proposed scheme as compared to the conventional method, resulting in the recovery of the equipment cost in less than a year.

## 1. Introduction

As the urban population is increasing, there are several challenges faced by large metropolitan cities. Some of these challenges include vehicle route guidance to avoid traffic jams [1,2], the effective utilization of the health care system [3,4], and efficient waste management [5,6]. The advancement in technology has provided several techniques and tools that can assist in solving these challenges, thus making way for smarter and cleaner cities [7,8].

Waste management is an important component of future smart cities [9,10]. The improper management of waste materials can be detrimental to the environment in many ways. This can significantly enhance land pollution and damage the soil, thus hurting human health and the ecosystem. Similarly, toxic materials can damage soil fertility leading to lower agricultural output. Waste mismanagement is also dangerous for marine animals and disrupts the supply of clean water to humans.

Waste materials are also a source of air pollution [11]. In particular, through burning waste materials, the respiratory health of humans can be badly affected [12]. Similarly, waste dumping can cause the release of methane-based greenhouse gas emissions [13,14]. This is one of the leading causes of climate change and global warming [15,16]. Other harmful effects of improper waste management include difficulty in extracting raw materials and damage to the animal habitat. Lastly, if the waste materials are not properly disposed of, it can lead to infectious diseases and other health issues.

Owing to the above issues, it is thus critical to design mechanisms for the efficient collection and disposal of waste materials [17,18,19]. Sustainable, eco-friendly, and technology-assisted strategies are needed to develop waste dumping systems. With the increase in population as well as the amount of waste generated from households and industries, it is also pertinent to design methods for time-efficient and effective waste collection [20,21,22,23,24].

The collection of waste relies on effectively using waste pickup trucks. Since the number of such waste pickup trucks is limited in number and the amount of waste is increasing day by day, managing the routes of these waste trucks and their pickup capacity are two critical issues. It is important to optimize the routes of waste pickup trucks so that cost-effectiveness and sustainability are achieved. For this purpose, technologies like the Internet of Things (IoT), Global Positioning System (GPS), and routing algorithms can be combined to formulate the best routes for waste pickup. The factors to be considered for waste vehicle routing include vehicle densities on the road and waste-related data. The goal is to select routes that reduce fuel consumption and maximize the disposal of waste.

The second issue is the management of the loading capacity of trucks that are used for picking up the waste. For better load management, the waste material type, waste priority, and truck capacity must be considered. Moreover, sensors can be installed, and regular waste fill-up data can be monitored to optimize the collection of waste. The efficient filling of waste pickup trucks is necessary to avoid extra trips and reduce fuel consumption. Moreover, it is vital to pick up waste of a critical nature and dump it in a time-efficient manner. Thus, the problem of waste pickup is essentially a resource management and allocation problem.

This paper focuses on the efficient allocation of waste material to waste pickup trucks. In this regard, a novel technique is proposed that uses the 0/1 knapsack algorithm to fill the waste pickup truck up to its loading capacity. The proposed algorithm takes into account factors such as waste volume, waste toxicity, and truck loading capacity, which are monitored using IoT sensors, and allocates waste to the trucks that maximize the waste utility while taking into account the truck’s loading capacity. The proposed technique was implemented in MATLAB software (version 2021) and compared against two other recent techniques from the literature. Simulation results show that the proposed technique improves the highly toxic waste collection by 47%.

The paper’s organization is as follows. Section 2 provides a review of related techniques and mechanisms. Section 3 describes the system model. The proposed technique is presented in Section 4. The implementation of the proposed work and evaluation of results is provided in Section 5. The conclusion is presented in Section 6.

## 2. Related Works

In this section, we describe the urban waste management system and its associated blocks. Moreover, we also present a review of recent work carried out related to urban waste collection. We also describe the novelty of the proposed technique compared to other available techniques.

### 2.1. Urban Waste Management System

**Figure 1:**
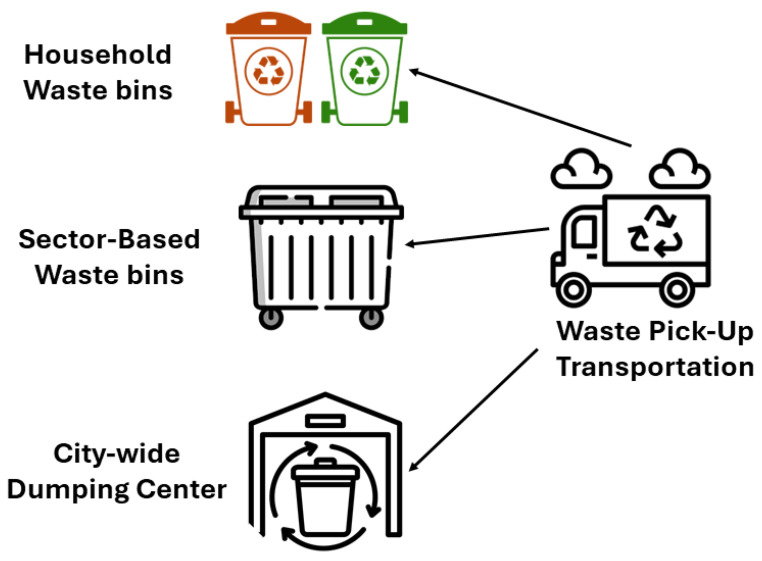
Urban waste management system.

The urban waste management system operates through a well-structured framework consisting of four pivotal blocks, as shown in Figure 1. Each block plays a crucial role in ensuring the efficient and responsible handling of waste throughout the city.

#### 2.1.1. Household Waste Bins

The first block is the waste bins at the houses. There are several types of bins installed in front of homes where citizens place their waste materials daily. There are separate bins for collecting general waste, glass materials, and recycled items.

#### 2.1.2. Sector-Based Bins

The second block of the waste management system is the sector-based bins placed at various geographical locations around the city. They are placed to cover small areas so that citizens can dump their waste in them at convenience. Sector-based bins serve as an intermediate collection point between the household bins and final disposal areas.

#### 2.1.3. Waste Pickup Transportation

The third block is the waste pickup trucks and transportation. These are assigned the duty of collecting city-wide waste from household bins and sector bins. The route of a pickup trucks is designed to maximize the collection of waste and reduce greenhouse emissions.

#### 2.1.4. City-Wide Dumping Centers

The last block of the waste system is the city-wide dumping centers where the waste is disposed of. These centers ensure the proper dumping of the waste and also use different methods based on the type of waste material. The dumping centers also manage if the waste is to be destroyed or recycled.

### 2.2. Literature Review

The work conducted related to waste collection focuses on classifying types of waste for better collection, the routing of pickup transportation for achieving efficient collection, and waste collection management. A summary of the literature review is presented in Table 1.

The work in [25] utilized pictures of waste to develop a method for identifying the type of material. The focus of the paper was on electronic equipment-type waste. The proposed framework requires users to upload images of the waste material to a central server. Two different types of neural networks are used for classification purposes using the images. The first algorithm is the convolution neural network that only identifies the type of electronic waste material. The second algorithm is the region-based convolution neural network that can further identify the size and category of electronic waste. The proposed technique achieves an accuracy of up to 97%.

In [26], a mechanism for the efficient routing of waste transport is proposed. The goal of the technique is to reduce the carbon emissions and associated costs of waste collection. The above problem was formulated as a mixed linear integer programming. Two optimization techniques, namely, fixed routing and variable routing, are used to achieve this task. For the fixed routing solution, the status of the waste bins is not known, and the route is planned according to the developed mathematical model. For the variable routing, the route is planned based on the updated status of the waste bins, which is communicated regularly to the server. Using the proposed mathematical model, the algorithm achieves reduced costs in terms of the waste transport travel distance and reduced toxic waste materials.

The work in [27] proposes a time and transportation capacity-based waste collection mechanism. The focus of the paper was to consider several practical parameters for waste collection. These include the capacities of both the waste trucks and waste disposal centers. Moreover, parameters such as the duration of collection shifts and closing times of shifts are also considered while making waste collection decisions. The proposed method also evaluates the number of waste trucks required for efficient collection. In addition, a method for efficient collection scheduling has also been proposed. The results highlighted the number of waste trucks required to meet all the objectives and related schedules of collection.

In [28], the work focused on textile-based waste collection. The textile waste is referred to as thrown-away clothing items. The key idea is to utilize sensors installed on the bins to regularly monitor the textile waste. The system was developed using an Arduino microcontroller and cheap sensors. The developed system was used to obtain real-time data on the waste bins. Based on the collected data, a dynamic selection of routes was proposed by the authors. The results showed reduced costs of waste collection in terms of high amounts of collected waste and reduced carbon emissions.

The work in [29] proposed an IoT-based system for efficient waste collection. The major idea in the work was to optimize the route of waste collection vehicles. Since the waste collection involves a variety of parameters, a Technique for Order of Preference by Similarity to Ideal Solution (TOPSIS)-based solution was proposed. The proposed work considered factors such as waste toxicity, waste volume, and waste generation time. The proposed technique improves the amount of collected waste while minimizing the distance traveled by the waste vehicles.

## 3. System Model

In this work, we considered multiple waste points located in different metropolitan locations, where different types of waste are collected. There are three waste bins in each of the waste locations carrying three different toxicity levels of waste material: high, medium, and normal toxicity levels. The bins will be filled by users based on their toxicity category; for example, if the waste is hazardous, it will be placed in the most toxic category bin. Similarly, general waste can be placed in the medium-toxic category bin. Lastly, the recyclable material can be placed in the least toxic category bin. It should be noted that classifying toxic category was not part of this study, and we rely on users to place the trash in the different bins. For classification purposes, IoT-based gas sensors can be used to measure the toxicity level of waste [30] to measure the hazard level of the waste for human health [31,32].

**Figure 2:**
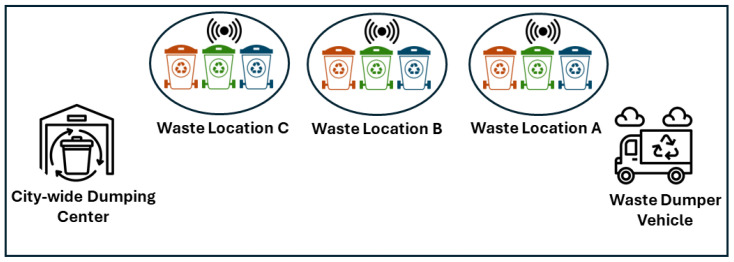
Waste collection scenario.

The waste bins located throughout the metropolitan area were of the same size and differentiated with different colors. Bins in each location were equipped with IoT sensors to monitor the filled capacity of the bin and the time since the bin had been emptied or replaced. IoT-based ultrasonic sensors were placed to measure the volume of waste in each bin [33]. All of the waste from different locations was collected by a dumper and dumped in a main garbage area. The dumper had a limited bin-carrying capacity and space to place a specific number of complete bins in it. It was supposed that the bins will be replaced with empty bins. All bins were supposed to be of the same size. A system model of the garbage collection is shown in Figure 2.

Suppose that each of the waste collecting bins is categorized as $B_{H}$, $B_{M}$, or $B_{N}$ for highly toxic waste, medium-toxic waste, and normal-toxic level waste material, respectively. The volume of each of the waste collection bins is *V*. If waste collected in the ith high-toxic bin is $V_{H}^{i}$, ith medium-toxic bin is $V_{M}^{i}$, and ith normal-toxic bin is $V_{L}^{i}$, and there are *N* high-toxic bins placed at the different locations of the metropolitan area, then the total volume of the high-toxic waste ($TV_{H}$), the total volume of the medium-toxic waste ($TV_{M}$), and the total volume of the normal-toxic waste materials ($TV_{L}$) are calculated as follows:

(1) $$TV_{H}=\sum_{i=1}^{N}V_{H}^{i}$$

(2) $$TV_{M}=\sum_{i=1}^{N}V_{M}^{i}$$

(3) $$TV_{L}=\sum_{i=1}^{N}V_{L}^{i}$$

The total waste material available ($\nu_{tot}$) from all the waste bins after a specific amount of time is the collective sum of all this waste:

(4) $$\nu_{tot}=TV_{H}+TV_{M}+TV_{L}$$

The dumper replaces these bins after a certain time interval of the day. Suppose a dumper has a bin-carrying capacity for placing *C* bins. If it picks up bins placed at the different locations of the areas up to their maximum capacity with *X* high-toxic bins, *Y* medium-toxic bins, and *Z* normal-toxic bins, then the total amount of waste collected by a dumper ($\eta_{tot}$) in its route is calculated as follows:

(5) $$\eta_{tot}=\sum_{i=1}^{X}\sum_{j=1}^{Y}\sum_{k=1}^{Z}V_{ijk}$$

The toxicity of waste depends on the waste material as well as the duration since it was placed there. As more time lapses, the toxicity of the waste also increases. The toxicity values of a unit amount of the highly toxic, medium-toxic, and normal-toxic bins after a certain time are represented as $T_{H}$, $T_{M}$, and $T_{N}$, respectively, and the waste collected after a certain time *t* is measured as *x* amount of highly toxic, *y* amount of medium-toxic, and *z* amount of normal-toxic waste. The toxicity values of waste collected from high-toxic bins ($\zeta_{H}$), medium-toxic bins ($\zeta_{M}$), and normal-toxic bins ($\zeta_{L}$) after time *t* are calculated as follows:

(6) $$\zeta_{H}=\sum_{i=1}^{X}x\times T_{H}$$

(7) $$\zeta_{M}=\sum_{i=1}^{y}x\times T_{M}$$

(8) $$\zeta_{L}=\sum_{i=1}^{z}x\times T_{L}$$

The toxicity level of all the collected waste bins ($\zeta_{tot}$) from the different toxic level bins after a certain time *t* is calculated as follows:

(9) $$\zeta_{tot}=\sum_{i=1}^{X}x\times T_{H}+\sum_{i=1}^{y}x\times T_{M}+\sum_{i=1}^{z}x\times T_{L}$$

## 4. Proposed Technique

Waste collection is a prime objective in any metropolitan area because it creates hazards for the human body and causes many infectious diseases. Collecting waste from different locations of a metropolitan area in a cost-effective way is a major challenge. In this work, an efficient waste collection mechanism is proposed to maximize the toxicity of the collected waste products within the maximum capacity of a dumper. The main features of the proposed scheme are mentioned below:

1. Calculate the optimum number of dumpers required to collect the waste bins.
2. Efficiently fill the dumpers with waste bins to enhance the toxicity of the collected waste material.

### 4.1. Calculating the Dumper Requirement

The dumpers have a specific bin-carrying capacity, and they are placed in diverse areas of a metropolitan area. The IoT sensors provide the live data of the empty bins placed in each area. A bin must be emptied if its waste overflows before the next waste collection. Suppose that there are *F* bins that may overflow if they are not emptied in the current collection, and D−C is the dumper capacity available for carrying waste bins, then the number of dumpers $D_{N}$ required to collect the bins placed at different locations is calculated as follows:

(10) $$D_{N}=\left\lceil \frac{F}{D_{C}}\right\rceil$$

### 4.2. Optimum Waste Collection Algorithm

The proposed scheme fills the dumper capacity with the different types of waste bins so that the maximum number of toxic bins are collected from different locations. The proposed scheme fulfills this requirement by applying a 0/1 knapsack algorithm. The knapsack picks items from a set of multiple items in such a way that the value of the picked items is optimal within its carrying capacity. Our problem is similar to the knapsack problem as in this problem, only waste bins that fall within the capacity of the dumper are required to be picked up from the different locations to maximize the toxicity level of the waste material present in the picked bins.

Our proposed scenario is analogous to the knapsack problem in all respects, as illustrated below and also mapped in Table 2.

The problem is mapped as follows:

1. The carrying capacity of the sack in our problem is the dumper capacity to place waste bins.
2. The items to be selected in the knapsack problem are the waste bins placed at different locations in the metropolitan area.
3. The weight of an item in the knapsack problem is identical to the waste bin, and it is uniform with value 1.
4. The value of an item in the knapsack problem is replaced with the priority of the waste bin that is required to be collected. The priority of a bin directly depends on the toxicity type of the waste material, the remaining capacity of the bin, and the time since the last bin was emptied. Suppose that each bin has a waste capacity of *K* and the *M* amount of the bin is filled with the same toxic type of material. If the waste is placed in the bin in the last duration *t*, then the priority of the bin *i* ($P_{bi}$) is calculated as follows:
  (11) $$P_{bi}=2^{\frac{k}{k-M}}\times t\times \zeta_{X}$$
  where $\zeta_{X}$ is the toxicity level of the waste material.

The 0/1 knapsack is an optimization algorithm, and it optimizes the priority of picked bins placed at different locations. To achieve this optimization, the capacity of the dumper is one of the major constraints in this problem. If the capacity of the bin-collecting dumper is represented by $D_{C}$ and there are N bins, then the collected number of bins $C_{b}$ that are picked is a constraint of this problem and is expressed as follows:

$$\sum_{i=1}^{N}C_{bi}\leq D_{C}$$

The optimization is achieved with the help of the following expression. Suppose that there are *N* bins and the priority value of the ith bin is $P_{bi}$, then the optimization of our problem by applying the 0/1 knapsack is expressed as follows:

$$Max\sum_{i=1}^{N}P_{bi}$$

To minimize the computing time, the 0/1 knapsack problem is solved using the tabular method instead of using dynamic and set-solving methods. The filling of the knapsack table along with the selection of the optimal waste bins are shown in Algorithm 1. The proposed knapsack algorithm is implemented by filling a knapsack table to scrutinize the number of bins placed at the different locations of a metropolitan area. A complete 0/1 knapsack algorithm in our proposed scheme is shown in Algorithm 1.
**Algorithm 1:** Waste Bin Collection Criteria
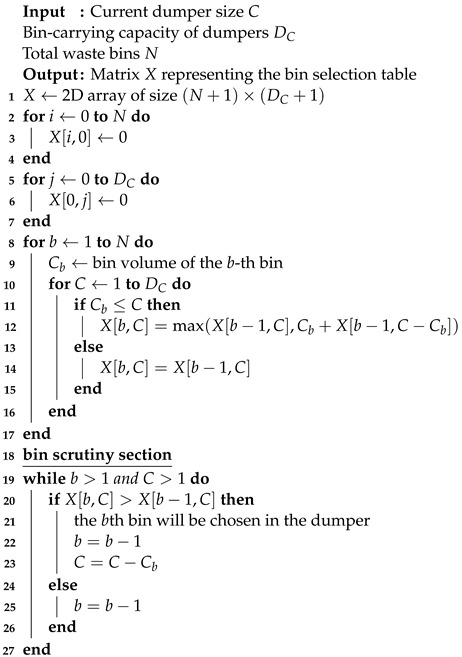


## 5. Results

In this section, we will present some of the results that were attained while simulating the waste collection based on our proposed mechanism. We labeled the high-, medium-, and normal-toxicity bins with numbers 10, 5, and 2, respectively. We chose the time since the last time the bin was collected from a set {1,2,3,…,10} through a uniform distribution. Similarly, we chose the weight/volume from the set {1,2,3,4,5}. For the simulations, we set the capacity of the vehicle to be equal to 10 bins. If the number of critical-waste bins was greater than the capacity of the vehicle, then another vehicle was sent. The number of critical-waste bins was the number of bins that had a priority value greater than the threshold value, which in our case, was equal to 100. The number of bins was set to be 40, i.e., 40 high-toxicity bins, 40 medium-toxicity bins, and 40 low-toxicity bins.

**Figure 3:**
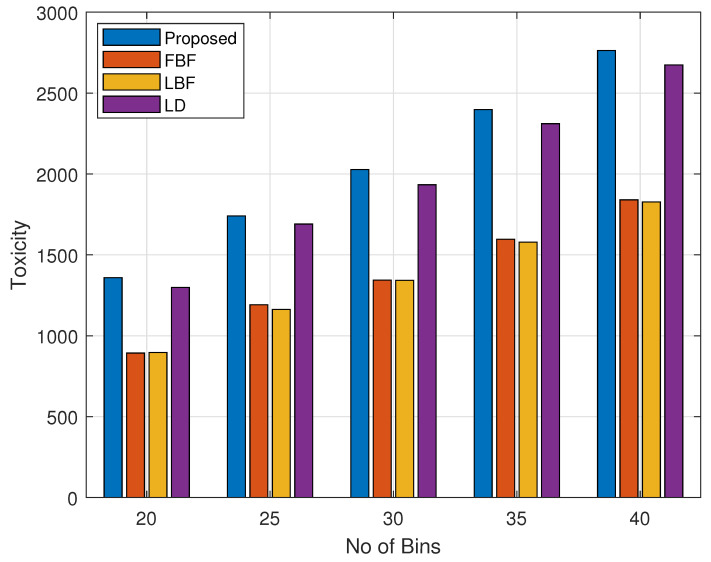
Collected waste toxicity levels vs. bin count.

Figure 3 illustrates the total toxicity value of the waste collected, showing the impact of deploying varying numbers of waste bins. Our proposed method was compared with three strategies: first bin first (FBF), which prioritizes bin collection based on location; largest bin first (LBF), which plans routes focusing on the largest bins nearing their capacity, assigning them a higher collection priority; and longest delay (LD), which grants higher priority to bins with longer collection delays. The capacity of the vehicle for this figure was set to be equal to 10 bins. The number of bins was increased from 20 bins to 40 bins in a step size of 5. From Figure 3, we can see that the scheme yielded the highest value of the overall toxicity for all values of the number of bins. Secondly, we can see that the value of toxicity increases from 1350 to 2700 for the proposed scheme when the number of bins was increased from 20 bins to 40 bins. The longest-delay scheme performs better as well. This is because the toxicity value depends on the time delay as well, and a larger delay value contributes to a higher toxicity value, as can shown in Equations (6)–(8). The FBF and LBF schemes performed the worst, as both location and volume do not have any involvement in the toxicity calculations.

**Figure 4:**
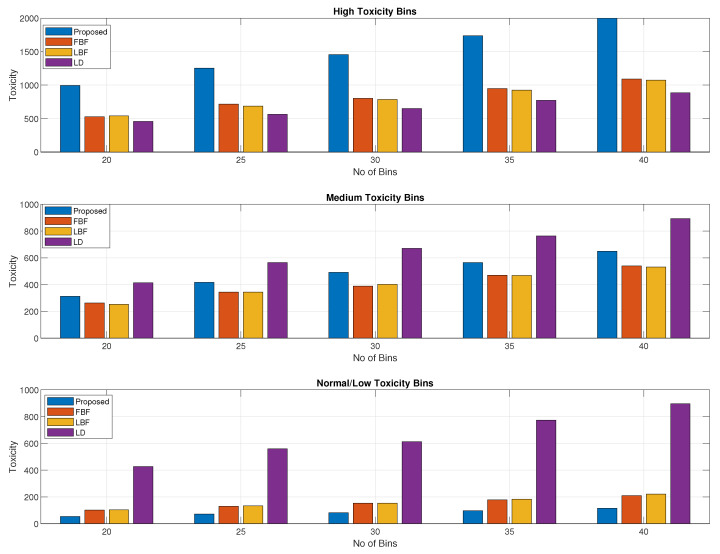
Toxicity levels of high-, medium-, and normal-toxicity bin waste collected vs. bin count.

Figure 4 shows the distribution of the waste collected based on high-, medium-, and normal-toxicity waste bins. The parameters for this figure are the same as in Figure 4. In this figure, we can see that the proposed scheme performed best for the high-toxicity waste bins, while the medium-toxicity waste bins were also comparable to those of the LD scheme. The normal-toxicity waste bins were collected in small proportions due to their priority value being in the low region. The normal-toxicity level bins would be collected with a longer delay as the material that is present in the bin does not become toxic rapidly with time.

**Figure 5:**
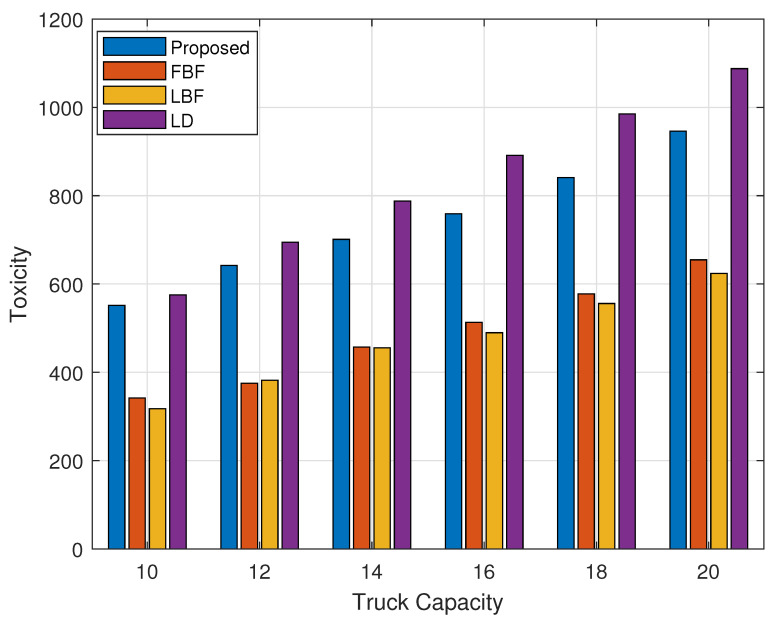
Collected waste toxicity levels vs. garbage truck capacity.

Figure 5 shows the results of the collected waste toxicity versus the truck capacity. The number of bins of each type was set to be equal to 40 bins. In this figure, we can see that the toxicity value of the collected waste is proportional to that of the LD scheme. The LD scheme is directly based on the parameter that defines the toxicity value of the waste, which is why it achieves a better performance in this case. It is important to mention here that as in the case of Figure 3, Figure 5 considers the overall toxicity value of all the waste bins combined. We can see that the FBF and the LBF schemes achieve a lower toxicity value of the collected waste as they do not directly consider the toxicity of the waste.

**Figure 6:**
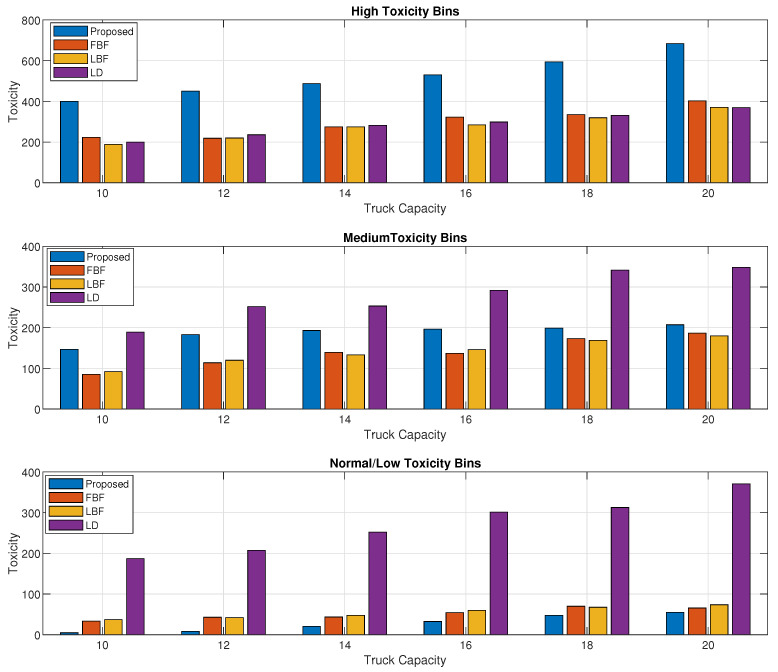
Toxicity levels of high-, medium-, and normal-toxicity bin waste collected vs. garbage truck capacity.

Figure 6 is the breakdown of the results from Figure 5 into high-, medium-, and normal-toxicity bins collected, and its overall toxicity in the subset. We can see that although the LD scheme was performing better in Figure 5, the amount of high-priority waste being collected by the proposed scheme is way better than in any of the schemes, including the LD scheme. For medium-toxicity bins, the proposed scheme achieves comparable results, and for normal-toxicity bins, the LD scheme achieves the highest value of the overall toxicity waste being collected.

**Figure 7:**
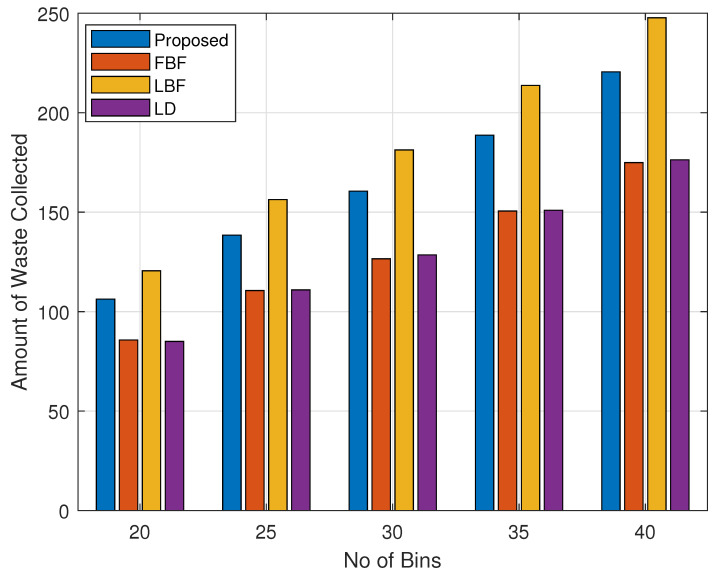
Collected waste volume/weight vs. bin count.

**Figure 8:**
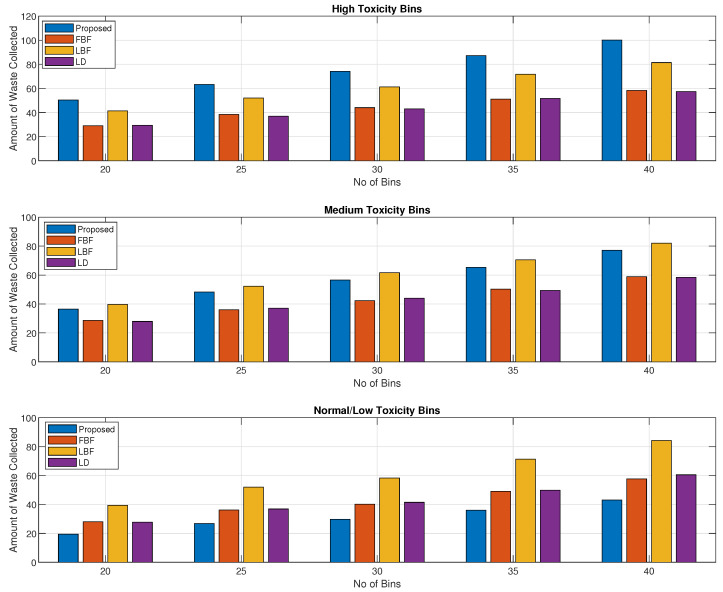
Volume/weight of high-, medium-, and normal- toxicity waste bins collected vs. bin count.

**Figure 9:**
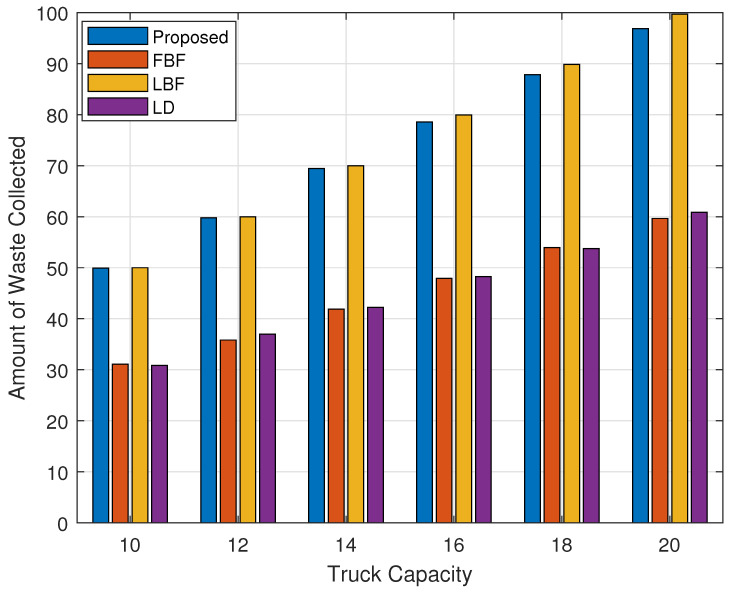
Collected waste volume/weight vs. garbage truck capacity.

**Figure 10:**
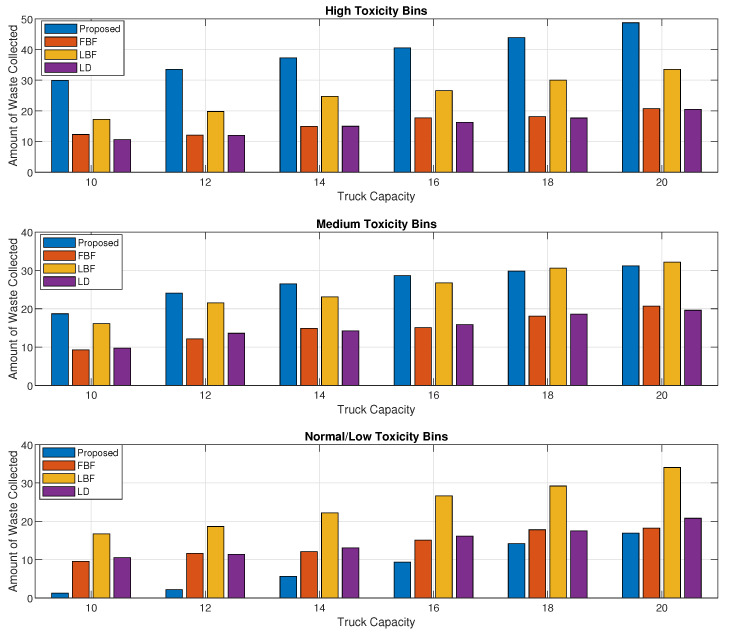
Volume/weight of high-, medium-, and normal-toxicity waste bins collected vs. garbage truck capacity.

From Figure 7, Figure 8, Figure 9 and Figure 10, we show the results for the amount of waste that was collected for various scenarios. For this set of figures, we kept the same values of the parameters as discussed in the discussion for Figure 3, Figure 4, Figure 5 and Figure 6. Figure 7 shows the amount of waste collected with a varying numbers of bins, which increases from 20, in a step size of 5, to 40. In Figure 7, we can see that the amount of waste collected increases with the increased number of bins. We can see that our proposed mechanism collects waste in an amount that is comparable to the largest-waste-bin-first scheme, while our proposed performs better than the FBF and the LD schemes. The downside of the increased value for the LBF scheme is that it achieves a lower value of toxicity, as can be seen from the trends shown in Figure 3, Figure 4, Figure 5 and Figure 6. The LD scheme was performing well for toxicity values, as is shown in Figure 3, Figure 4, Figure 5 and Figure 6, but in terms of the amount of waste collected, it performed poorly, as shown in Figure 7. Meanwhile, the FBF scheme performed worst in all the cases because the waste collection was not conducted on any specific criteria, and so the scheme acts like random bin collection, as it will be collecting bins that come in its way.

A breakdown of the values in Figure 7 in terms of the high-, medium-, and normal-toxicity bins is shown in Figure 8, where we can see that for high-toxicity bins, the proposed method performs better than any of the other schemes. The performance in the medium-toxicity waste bin collection is also better than those for the LBF and LD schemes, while the LBF scheme achieved a slightly better value for the medium-toxicity waste bins and achieved the best value for the low/normal-toxicity waste bins. The results in Figure 4 and Figure 8 confirm that our proposed method achieves a higher priority for the high-toxicity waste bins while the medium- and the normal-toxicity waste will grow in priority if it is delayed by a large value or the bin is about to be filled.

Figure 9 shows the amount of waste collected versus the carrying capacity of the vehicle which was increased from 10 to 20 in a step size of 20. The numbers of bins for Figure 9 and Figure 10 were set to be equal to 40. From Figure 9, we can see that the amount of waste collected increases with the increased capacity of the truck. We can see that the value increases from 50 to about 97 for the proposed scheme when the capacity of the truck is increased from 10 to 20. The LBF scheme performance is neck-in-neck with that of the scheme in terms of the amount of waste collected for the lower capacity of the truck and achieves slightly higher when the capacity of the truck is greater than 15. The FBF and the LD schemes perform worst as they do not rely on the amount of waste for calculating their bin collection priority. LBF collects bins based on their weight/volume so that is why it achieves a higher value for the amount of waste collected, but it performs poorly when we consider the toxicity measure of the collected waste, as can be seen in Figure 5 and Figure 6.

When we break down the results in Figure 9 to show the amount of waste collected from the high-, medium-, and low/normal-toxicity waste, as shown in Figure 10, we can see that the proposed method performs significantly better than the LBF scheme while it performs multi-fold higher than the FBF and the LD scheme for the reasons that were discussed in the explanation of Figure 9. The proposed scheme performs better than the LBF scheme for the medium-toxicity waste bins as well and only performs poorly for the low/normal-toxicity waste bins as only a small set of the normal-toxicity waste bins made it into the high-priority zone due to a longer wait time or due to the amount of waste that might become full in a short time.

### Cost Analysis of the Proposed Method with Legacy System

Our proposed method optimizes waste collection and reduces the number of required trips by deploying an IoT system. This section presents a cost analysis of the proposed system, demonstrating that over the long term, the initial investment in IoT deployment will prove beneficial compared to the legacy method. For this analysis, we assumed that the vehicles are already available, so the capital cost of the legacy system is not considered. We considered that there are N bin locations, each with three bins. The distance between two bin locations was set at 100 m; thus, if there are 100 bin locations, our vehicles would need to travel approximately 10 kilometers for each trip. For the legacy systems, we estimated a per-kilometer cost of USD 20, 30 and 40, encompassing fuel, maintenance, salaries, and other associated expenses in waste collection.

**Figure 11:**
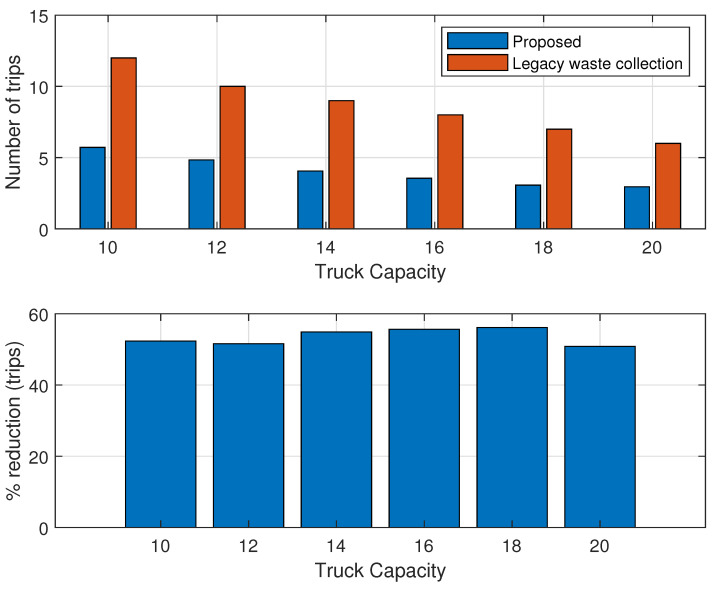
Number of trips required (comparison).

Regarding the proposed system, we evaluated the improvements in terms of the reduced number of trips required, as illustrated in Figure 11. The analysis indicates a clear reduction of at least 50% in trips required for the optimized system compared to those of the legacy systems.

**Figure 12:**
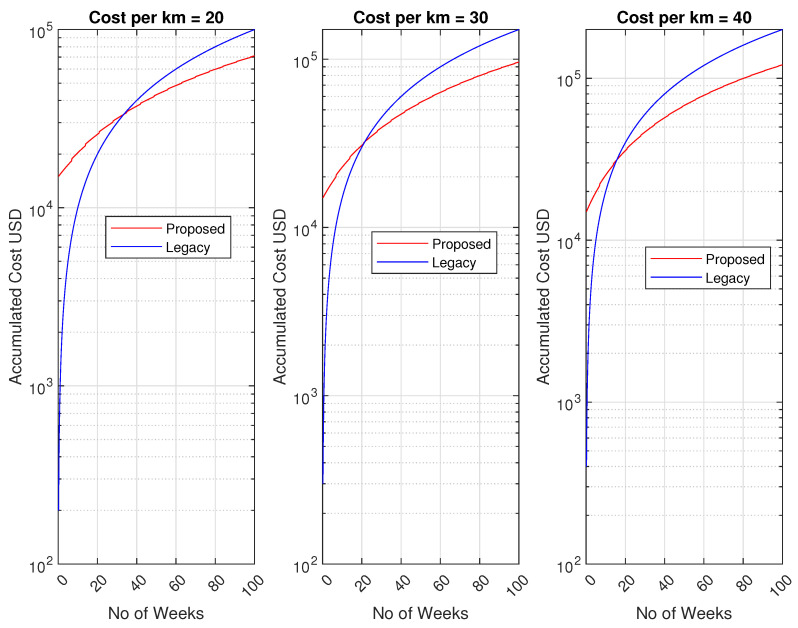
Cost analysis.

Turning to the additional costs for the proposed system, we considered that each bin is equipped with an ultrasonic sensor, priced at around USD 50. These sensors are WiFi-enabled and can utilize the WiFi network available at the end-user premises. Additionally, we anticipated a maintenance cost of approximately 1% of the total sensor costs per week. Furthermore, there will be an operational cost, estimated at 2% of the total cost of all the sensors per week. In assuming there will be five trips per week, the cost analysis is depicted in Figure 12.

Referring back to the proposed algorithm, as indicated in Figure 11, we observed that the number of trips will be halved compared to the legacy system. Consequently, the cost per week of operation will also be reduced by half. Although the proposed model entails higher initial costs due to sensor installation, the weekly operational expenses will decrease. It is evident that the accumulated costs will break even at approximately 30 weeks for the case where the operational cost is 20 USD per km, while it will break even at 20 and 17, when the cost per km is USD 30 and 40, respectively.

## 6. Conclusions

Efficient waste collection is an important part of waste management systems. Waste collection faces challenges such as routing waste dumper vehicles and filling their capacity to maximize the collection of toxic waste. In this study, we developed an optimal waste collection algorithm that loads the dumper vehicle with waste bins across a city to maximize the toxicity of the collected waste bins while meeting the dumper capacity requirements. The given problem was solved using the 0/1 knapsack algorithm where the sack capacity is taken as the bin-carrying capacity of the dumper, the weight of an item is taken as a waste bin, and the value of an item is taken as the priority of the bin that depends on the toxicity and the duration of the waste. The proposed technique was implemented in a MATLAB simulator, and the simulation results verify the significance of the proposed solution in terms of high-priority toxic waste collection compared to other techniques in the literature. Furthermore, the cost analysis shows that the cost of the equipment installed at each waste disposal premise will be covered in less than a year.

## Figures and Tables

**Table 1:** Literature review summary.

Reference	Goal	Technique	Results
[25]	Waste classification	Image processing Electronic waste User image capturing CNN R-CNN	97% accuracy
[26]	Waste vehicle routing	Cost optimization MILP problem Fixed routing Variable routing	Reduced travel distance Reduced carbon emissions
[27]	Waste collection	Practical parameters Waste truck capacity Disposal center capacity Shift duration Shift closing times	Scheduling of waste trucks Number of trucks
[28]	Waste collection	Textile waste considered Sensor-based bins Arduino-based solution Real-time data collection Dynamic route selection	Increased waste collection Reduced carbon emissions
[29]	Waste vehicle routing	IoT-based solution TOPSIS-based routing Waste toxicity Waste volume Waste generation time	Increased waste collection Reduced travel distance

**Table 2:** Knapsack mapping parameters.

	Bin Selection Parameters	Knapsack Parameters
$D_{C}$	Bin placement capacity of the dumper	Item-carrying capacity of sack
$C_{b}$	Waste bin	Weight of item
$P_{b}$	Priority level of the collected bin	Value of item

## Data Availability

All data are available in the paper.
